# Molecularly Engineered Dual-Emission Pathways with Monomer–Excimer Interplay for Single-Component Blue and White Organic Light-Emitting Diodes

**DOI:** 10.1021/acsaom.6c00161

**Published:** 2026-05-27

**Authors:** Ehsan Ullah Rashid, Rishika Suresh, Dmytro Volyniuk, Sathiyanarayanan Kulathu Iyer, Juozas V. Grazulevicius

**Affiliations:** † Department of Polymer Chemistry and Technology, Faculty of Chemical Technology, 70309 Kaunas University of Technology, K. Baršausko g. 59, LT-51423, Kaunas, Lithuania; ‡ School of Advanced Sciences, 30026 Vellore Institute of Technology University, Vellore 632 014, India

**Keywords:** tetrahydrodibenzophenanthridine, single-component white emission, white emissive aggregates, white OLED, excimer emission

## Abstract

The design of an organic molecule that inherently exhibits multiple emissive channels provides an elegant strategy for structurally simple yet spectrally broad white organic light-emitting diodes (OLEDs). Herein, we report the rational design and synthesis of a donor−π-acceptor luminophore (**DBP-PXZ**), comprising a phenoxazine donor moiety and a tetrahydrodibenzo­phenanthridine acceptor fragment linked through a twisted phenylene bridge. The photoluminescence (PL) spectrum of the solid sample of the compound exhibits dual emission bands, resulting in single-component white emission with CIE coordinates of (0.31, 0.38). PL spectral analysis of the solid solutions in polar host matrices and in a PMMA matrix demonstrates that controlled modulation of intermolecular proximity selectively activates or suppresses a low-energy emissive channel. Close chromophore packing activates intermolecular charge transfer (CT)-type excimer emission, while spatial isolation suppresses this pathway and yields purely blue emission from the monomeric CT state. Computational calculations support the stabilization of an intermolecular CT excited state of dimeric models relative to the monomeric ones. Leveraging this dual-emissive nature, host-free OLEDs based on a thin layer of **DBP-PXZ** as emissive layer exhibit a broadband electroluminescence spectrum spanning the range of ca. 385–800 nm. The device with an emissive layer of diluted **DBP-PXZ** with a wide-bandgap host effectively suppresses excimer formation and enables a pure blue EL spectrum. This work highlights **DBP-PXZ** as a single-component emitter enabling tunable blue-to-white emission via monomeric and excimer CT states, offering a compositionally simplified and heavy-metal-free emitter approach inspired by a sustainability-driven OLED design.

## Graphical abstract

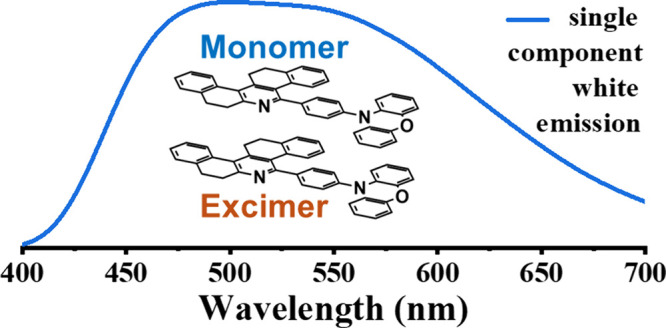

## 1 Introduction

Single-component white emitters have emerged as compelling organic materials for advanced white organic light-emitting diodes (WOLEDs), offering a transformative alternative to conventional device structures that rely on multiple emissive layers.^1,2^ The conventional WOLED designs are typically based on the separated red, green, and blue (RGB) emitting layers^2,3^ or on stacked emissive layers with complementary emissions (e.g., blue combined with yellow/orange).^4,5^ While the implementation of these approaches can result in achieving high color quality, these approaches inevitably introduce structural complexity of the devices, interfacial exciton losses, color instability, and increased fabrication costs.^6^ An alternative strategy involves co-dispersion of two complementary emitters (blue and yellow/orange) within a single emissive layer to achieve nominally single-layer white emission. However, this approach remains inherently multicomponent and is governed by inter-emitter interactions and complex energy-transfer dynamics.^7,8^ Chen et al.^9^ reported a warm-white OLED based on an active monolayer comprising dibenzofuran and a carbazole derivative of diphenyl triazine exhibiting blue thermally activated delayed fluorescence (TADF). It served as a blue emissive host. The phenoxazine derivative of dipyridophenazine exhibiting orange-red TADF was used as a dopant. The device showed an external quantum efficiency (EQE) of 32.8%, CIE coordinates of (0.41, 0.46), and a low turn-on voltage of 2.6 V.^9^ Despite the proficient device performance achieved with host–dopant mixed emissive layers, such devices intrinsically suffer from exciton redistribution and concentration-dependent energy transfer processes, which frequently lead to color instability and, most importantly, increased fabrication complexity and cost.^10^ In contrast, harvesting of multiple emissive states within a single molecular framework enables white emission to be obtained from a single emissive layer, effectively eliminating interfacial energy barriers, suppressing exciton quenching, and substantially simplifying device structures.^1,11^ The strategies toward single-component white emission have been reported, encompassing intriguing photophysical phenomena including dual fluorescence,^12,13^ fluorescence and phosphorescence,^14^ and dual phosphorescence from the radiative relaxation of two distinct triplet excitons followed by non-Kasha phosphorescence.^15^ Dual-fluorescent organic fluorophores, exhibiting high-energy monomer emission and low-energy excimer emission, have been explored for single-component white emission.^16,17^ However, achievement of broadband single-component white emission remains challenging, as the delicate balance between high-energy monomer emission and low-energy excimer emission is highly sensitive to various intrinsic and extrinsic physicochemical factors, often leading to dominance of low-energy emission and spectral imbalance. Excimer denotes an excited-state complex between identical molecules, whereas exciplex is an excited state complex formed between spatially separated donor and acceptor molecules via intermolecular interactions.^18^ Owing to spatial separation of electron (on acceptor) and hole (on donor) density, exciplexes often exhibit TADF benefiting from a sufficiently small single–triplet gap.^19^ Li et al.^20^ reported on a highly twisted molecular system comprising a triphenylamino donor moiety, a pillar[5]­arene bridge, and a triphenylacrylonitrile acceptor fragment, in which the pillar[5]­arene induces strong donor–acceptor decoupling. The emitter displayed dual emission, with blue light originating from a monomeric locally excited (LE) state and yellow emission arising from the intramolecular through-space charge-transfer (TSCT) state. Balanced white emission (CIE coordinates of (0.30, 0.36)) was achieved only by externally modulating the microenvironment via a DMF/glycerol viscosity gradient, highlighting the sensitivity of emission balance to environmental conditions. Lee et al.^21^ reported a dual-emissive luminophore comprising a diphenyl triazine acceptor and benzofurocarbazole donor units, further functionalized with a conjugation-broken diphenothiazine-benzene moiety. Blue emission originated from intramolecular CT between the benzofurocarbazole donor moiety and the diphenyl triazine acceptor unit, whereas formation of an intermolecular excited-state complex between diphenothiazine-benzene and diphenyl triazine units generated a lower-energy yellow TADF. The neat films showed excimer emission and suppressed blue emission through efficient energy transfer.^21^ Consequently, white electroluminescence was achieved only at low dopant concentrations (5%) in a host, with CIE coordinates of (0.25, 0.31).

In this context, we introduce a donor−π-acceptor type luminophore (**DBP-PXZ**) comprising a phenoxazine donor moiety linked via a phenylene bridge to a tetrahydrodibenzo­phenanthridine (DBP) acceptor fragment.^22^ Excimer emission from the derivative of phenoxazine was previously reported. The diphenyl sulfone derivative of phenoxazine showed excimer emission. The enhanced short-range intermolecular interactions, revealed by crystal structures, facilitated excimer formation. This short-range interaction derived excimer emission was not observed for the analogous diphenyl sulfone derivative of an acridine donor.^22^ In contrast, derivatives of the DBP acceptor exhibited only blue monomeric emission, with no prior reports of excimer states.^23,24^ However, N-heterocyclic frameworks emerged as privileged frameworks in organic optoelectronics^25^ owing to their modular syntheses, tunable frontier orbital energies, and inherent structural diversity.^1,26,27^ Many of the N-heterocyclic frameworks can be synthesized via concise or multistep protocols that align with green-chemistry synthesis approaches.^28–31^ Derivatives of N-heterocyclic frameworks have been widely explored as single-component white emitters.^32,33^ Furthermore, the derivatives of carbazole and pyridine exhibit blue fluorescence and low-energy excimer emission, resulting in single-component white light emission.^34^ White emission was also achieved from the derivatives of carbazole and diphenylamino-substituted phenylquinazoline.^35^ In these frameworks, acid-induced protonation of the acceptor enhanced intramolecular charge transfer, causing a red-shifted emission that coexisted with the simple blue emission. This allowed achieving dual-band white electroluminescence.^35^

In the present study it is shown that, due to the twisted D−π–A geometry of **DBP-PXZ**, it exhibits localization of the highest occupied molecular orbital (HOMO) on the phenoxazine unit and the lowest unoccupied molecular orbital (LUMO) on the acene-like DBP framework, establishing a well-defined intramolecular CT monomer state that shows blue fluorescence. In the solid state or in aggregated form, the electron-rich phenoxazine unit and extended DBP framework engage in specific short-range intermolecular interactions to generate a lower-energy excimer CT state that emits in the greenish-yellow spectral region. The balanced coexistence of these monomeric and excimer emissive channels enables intrinsic single-component white emission from the solid samples of **DBP-PXZ** with CIE coordinates of (0.31, 0.37). The DBP acceptor framework is critical in achieving balanced emissions. Its fused heterocyclic structure, disrupted conjugation by sp^3^-hybridized benzylic methylene units, results in the reduction of excessive planarity and modulates short-range intermolecular interactions. This allows cooperative excimer formation with the electron-rich phenoxazine donor while preserving monomeric blue emission, enabling intrinsic single-component white emission of **DBP-PXZ**. The key aspect of this work is a molecular design that deliberately balances two emissive channels within a single component, i.e., a blue emission from an intramolecular CT monomer and a lower-energy emission from excimer-like CT that becomes accessible only in the solid state. The quantum chemical calculations of monomer/dimer electronic structures were performed to analyze the nature of dual-emissive excited states. Combined steady-state and time-resolved PL spectroscopy data allow establishing that the white emission originates from controlled intermolecular contact formation, enabling single-component white electroluminescence without the need for multiemitter blending or engineered donor–acceptor exciplex interfaces.

## 2 Experimental Section

### 2.1 Instrumentation

Nuclear magnetic resonance (NMR) measurements were acquired on a Bruker Ascend 400 spectrometer to confirm the molecular structure. The samples were dissolved in CDCl_3_, and tetramethylsilane (Si­(CH_3_)_4_) was used as the internal chemical-shift reference for both ^1^H and ^13^C NMR analyses. The melting behavior was examined with an Electrothermal MEL-TEMP apparatus. Thermal endurance and phase transitions were evaluated under an inert nitrogen atmosphere using complementary thermogravimetric and calorimetric protocols: TGA was performed on a PerkinElmer TGA 4000 at a heating rate of 20 °C min^–1^, while DSC scans were collected on a PerkinElmer DSC 8500 at 10 °C min^–1^. UV–vis–NIR absorption spectra of dilute toluene and THF solutions, as well as vacuum-deposited neat films, were recorded with a PerkinElmer Lambda 950 spectrophotometer. Steady-state photoluminescence spectra and absolute photoluminescence quantum yield of dilute solutions, neat films, and molecularly dispersed solid-state films were obtained using an Edinburgh Instruments FLS980 spectrometer. Electrochemical measurements were carried out at 25 °C on an Autolab potentiostat–galvanostat in a three-electrode configuration. A 2 mm diameter carbon disk, platinum wire, and silver wire were employed as working, counter, and quasi-reference electrodes, respectively. Dry dichloromethane containing 0.1 M tetrabutylammonium hexafluorophosphate served as the supporting electrolyte; the analyte concentration was 10^–3^ M, and the scan rate was 50 mV s^–1^. The photoelectron emission spectra were recorded in air employing a 6517B Keithley electrometer, an ASBN-D130-CM deep UV deuterium light source, a CM110 1/8 m monochromator, and a personal computer installed with the appropriate software. The time-of-flight method was used for hole and electron mobility measurements using an EKSPLA NL300 Nd:YAG laser (third-harmonic of 355 nm and pulse duration of 3–6 ns), Keithley 6517B electrometer, and Tektronix TDS 3032C oscilloscope. TOF measurements were determined in air and at room temperature. The thickness of the vacuum-evaporated layer was measured using the Profilm3D pyrophotometer. All potentials were internally calibrated against the ferrocene/ferrocenium (Fc/Fc^+^) redox couple. For the fabrication of OLEDs, a vacuum chamber equipped with a multisource thermal evaporator was utilized to deposit high-quality organic thin films and metal electrodes under an ultrahigh vacuum of (2–5) × 10^–6^ mbar. The deposition was performed sequentially on a prepatterned indium tin oxide (ITO) glass substrate, which has a sheet resistance of 20 Ω/square and was provided by Ossila. The eight pixels with the active OLED area of 4.5 mm^2^ were obtained on each substrate. Before the OLED deposition, the ITO glass substrate was subjected to pretreatment with an oxygen plasma to enhance the work function and diminish the hole injection barrier. For measuring the electroluminescent characteristics of OLEDs, luminance–current density–voltage curves were recorded using a multisource meter (Keithley 2400), a spectrometer (Avantes AvaSpec-2048XL), a certified photodiode (PH100-Si-HA-D0) operated via the PC-based 11S-LINK power and energy monitor (from STANDA), and a computer equipped with the relevant software.

### 2.2 Computational Methodology

Organic compounds based on donor–acceptor type structures frequently generate CT excited states, presenting significant challenges in accurately describing their excited-state energies, electron density distributions, and intermolecular couplings. In such systems, the spatial separation of frontier orbitals often leads to a detailed balance between local excitation and CT character, making conventional hybrid functionals inadequate for reliable excited-state modeling.^36^ To achieve reliable predictions, long-range corrected hybrid functionals combined with an optimally tuned range-separation parameter (ω) are required. In this study, the electronic geometry of **DBP-PXZ** was optimized to the ground state using density functional theory (DFT) employing the ωB97XD functional^37,38^ with the 6-31G** basis set, using the Gaussian 16 program.^39^ The range-separation parameter ω was subsequently tuned for time-dependent DFT (TD-DFT) calculations following a nonempirical tuning protocol based on Koopmans’ theorem as stated in eqs 1 and 2.^40^

1 $$-E_{H}=IP=E_{0}^{+}-E_{0}$$

2 $$-E_{L}=EA=E_{0}-E_{0}^{-}$$

Here, *E*_H_ and *E*_L_ represent the energies of the HOMO and LUMO, IP and EA are ionization potential and electron affinity, while *E*_0_, *E*_0_^+^, and *E*_0_^–^ are the ground-state energy of neutral, cation, and anion electronic systems. To satisfy these conditions, ω was determined by minimizing the function *J* ^ω^, defined as a measure of deviation between orbital energies and their corresponding computed IP and EA (eq 3).^41–43^

3 $$J^{\omega }={\left[E_{H}^{\omega }+IP^{\omega }\right]}^{2}+{\left[E_{L}^{\omega }+EA^{\omega }\right]}^{2}$$

The conductor-like polarizable continuum model (CPCM) was employed to account for solvent polarization effects, with both toluene and tetrahydrofuran (THF) considered as dielectric media. Optimal ω values were determined for each solvent environment, yielding an ω of 0.039 Bohr^–1^ for toluene and an ω of 0.0013 Bohr^–1^ for THF. Since, excimer formation is also a crucial focus of this work, the dimer of **DBP-PXZ** was also optimized at the ωB97XD/6-31G** level. For the dimer, the optimal value of ω under CPCM/THF solvation was found to be 0.0007 Bohr^–1^. Natural transition orbital (NTO) analysis was subsequently performed to visualize electronic excitations.

## 3 Results and Discussion

### Synthesis

Compound **DBP-PXZ** was obtained in two steps (Scheme 1). First, 4-(10*H*-phenoxazin-10-yl)­benzaldehyde was synthesized via CuI-catalyzed Ullmann C–N coupling of phenoxazine with 4-bromobenzaldehyde in dimethylformamide with 43% yield. The subsequent condensation of this aldehyde with 3,4-dihydronaphthalen-2­(1*H*)-one in ethanol in the presence of ammonium acetate yielded **DBP-PXZ** as a pale-yellow solid in 10% yield. Full experimental procedures along with the corresponding ^1^H and ^13^C NMR spectroscopy data and mass spectrum of DBP-PXZ are provided in the Supporting Information (Figures S1–S5).

**Scheme 1:**
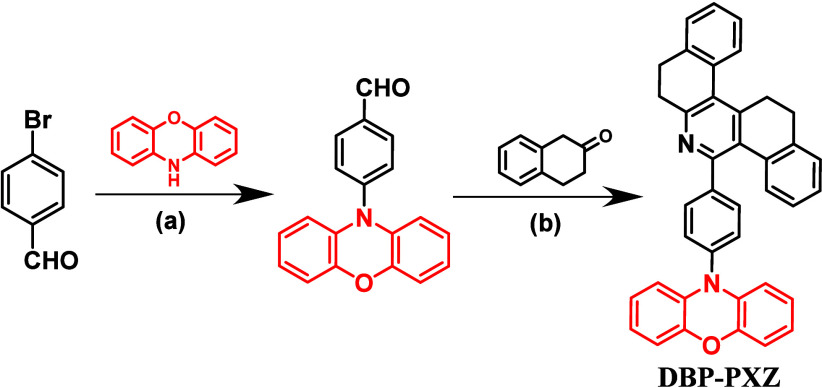
Synthesis Scheme of **DBP-PXZ** ^a^

a Conditions: CuI, K_2_CO_3_, DMF, N_2_, 100 °C (a); NH_4_OAC, EtOH (b).

### Optimized Geometry and Molecular Orbitals

Implementing the density functional theory (DFT) approach, the ground-level optimized geometry of **DBP-PXZ** at the ωB97XD/6-31G** level reveals a conspicuously twisted donor–acceptor structure (Figure 1). The DBP unit and the phenylene ring adopt a dihedral angle of ca. 49°, while the phenylene–phenoxazine twist is nearly orthogonal (ca. 76°). This high torsion effectively attenuates π-conjugation across the D−π–A framework and enforces spatial separation of the frontier orbitals. Hence, the HOMO is predominantly localized on the phenoxazine moiety, with only minor extension onto the phenylene ring, whereas the LUMO is largely confined to the DBP acceptor framework. This pronounced donor-localized HOMO and acceptor-localized LUMO pattern evidences a prominent intramolecular CT. Notably, the DBP fragment exhibits an extended, polycyclic framework reminiscent of archetypal acenes such as anthracene^44,45^ and perylene,^46^ which are well-known for their strong intermolecular π–π interactions and propensity to form excimers and other intermolecular interaction derived excited states.

**Figure 1:**
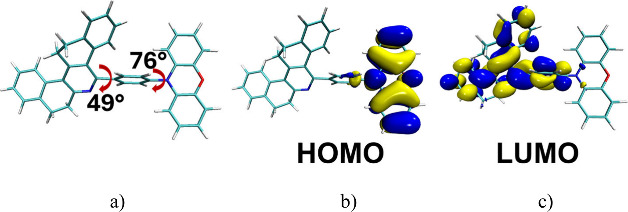
Optimized geometry (a) and the HOMO (b) and LUMO (c) of **DBP-PXZ** at the ωB97XD/6-31G** level.

### Thermal and Electrochemical Analysis

Thermal robustness is an essential prerequisite for the practical deployment of organic luminophores in long-lived optoelectronic devices. The thermal stability and morphological transitions of **DBP-PXZ** were studied by thermogravimetric analysis (TGA) and differential scanning calorimetry (DSC) (Figure 2a,b, Table 1). The 5% weight loss temperature (*T*_d_^5%^) of **DBP-PXZ** was observed at ca. 361 °C. DSC analysis showed a pronounced melting transition at 263 °C in the first heating scan. After the cooling scan, the glass transition at 122 °C was observed during the second heating scan of **DBP-PXZ**. High *T*_d_^5%^ and glass transition temperature underscore the suitability of **DBP-PXZ** for vacuum-deposited thin-film fabrication.

**Figure 2:**
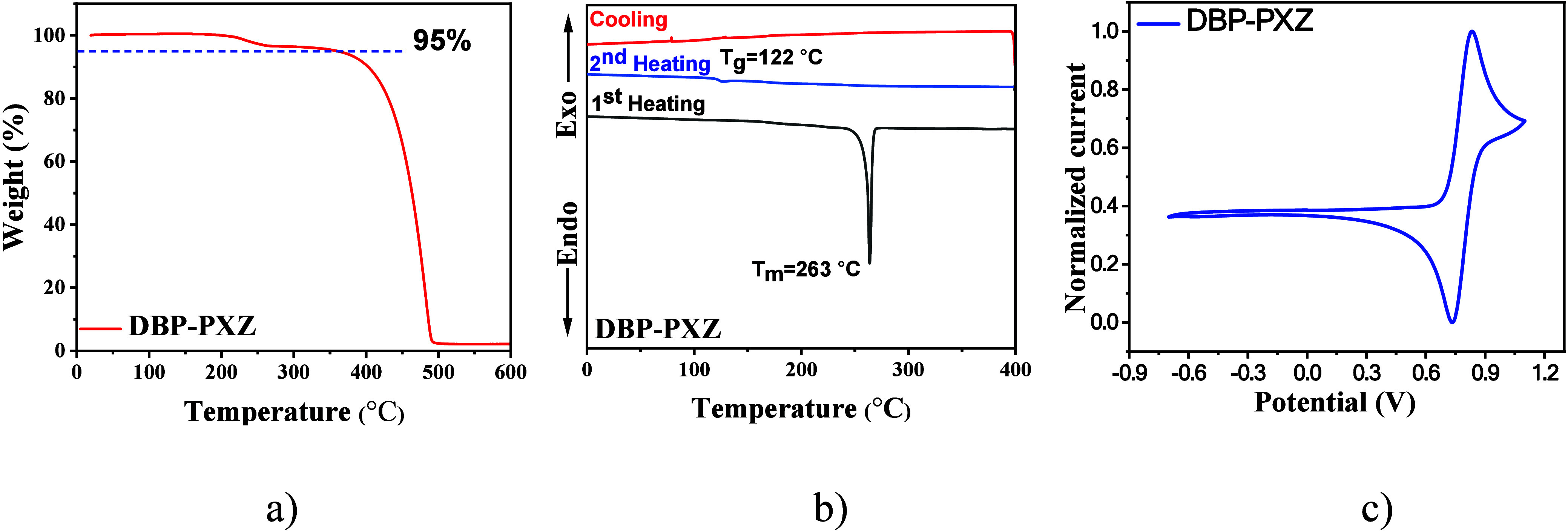
TGA (a), DSC (b), and CV (c) curves of **DBP-PXZ**.

**Table 1:** Thermal and Electrochemical Characteristics of **DBP-PXZ** ^a^

Compound	*T*_g_ (°C)	*T*_m_ (°C)	*T*_d_^5%^ (°C)	*E*_g_^opt^ (eV)	IP_CV_ (eV)	IE_PE_ (eV)	EA_PE_ (eV)
**DBP-PXZ**	122	263	361	2.95	5.81	6.0	3.05

a Glass transition temperature (*T*_g_) and melting temperature (*T*_m_), the 5% weight loss temperature (*T*_d_^5%^), optical bandgap (*E*_g_^opt^) measured from the onset of the absorption spectrum of the neat film, ionization energy (IP), cyclic voltammetry (CV), ionization energy (IE), photoelectron emission (PE) spectroscopy, and electron affinity (EA).

The redox characteristics and frontier energy levels of **DBP-PXZ** were elucidated by cyclic voltammetry (CV) of a dichloromethane solution of **DBP-PXZ** and photoelectron emission (PE) spectroscopy of a vacuum-deposited thin film of **DBP-PXZ**. The CV voltammogram exhibited a well-resolved and reversible oxidation scan with an onset of oxidation potential of 0.71 V versus Fc/Fc^+^ (Figure 2c). Using the standard relationship,^47^ IP_CV_ = +5.1, the corresponding ionization energy (IP_CV_) was determined to be 5.81 eV (Table 1 and Figure 3a).

**Figure 3:**
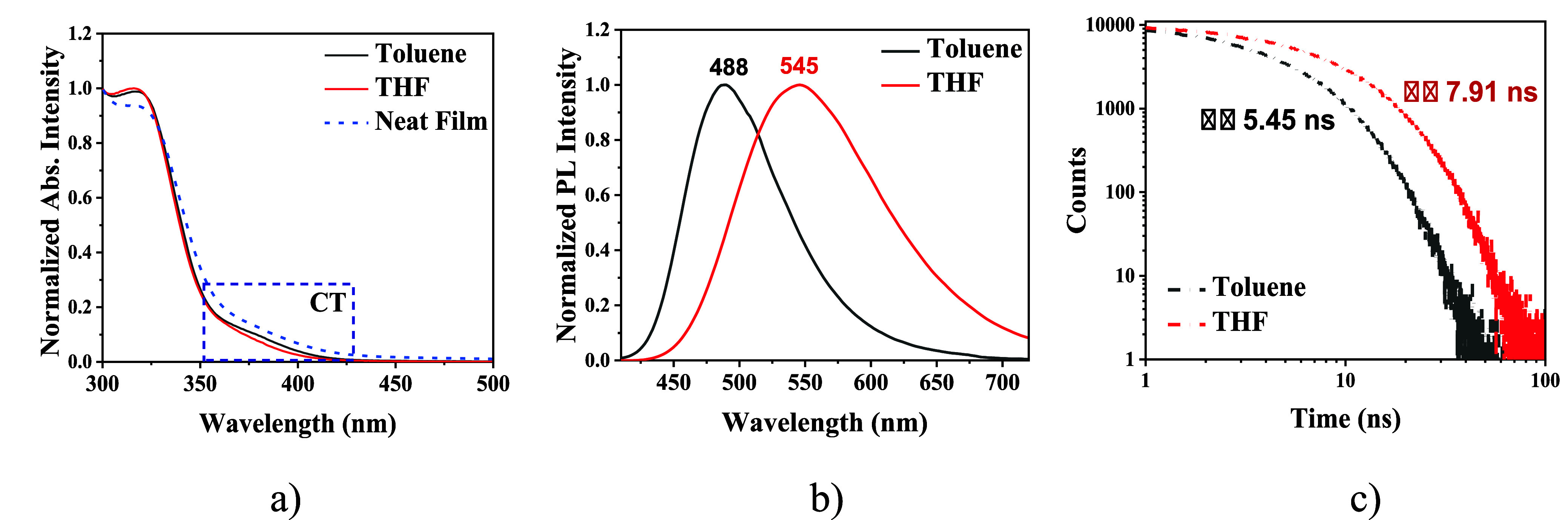
UV–vis absorption spectrum of **DBP-PXZ** (a); steady-state PL spectra of toluene and THF solutions (b); and time-resolved PL decay curves of toluene and THF solutions of **DBP-PXZ** (c).

### Photophysical Properties

#### Photophysical Properties of Solutions

The UV–vis absorption spectra of toluene and THF solutions of **DBP-PXZ** (Figure 3a) display closely analogous dual absorption bands, consistent with a separation between locally excited and charge-transfer manifolds. A pronounced high-energy band at ca. 318 nm is assigned primarily to a π–π* transition localized on the conjugated molecular framework. The weaker lower-energy shoulder extending across 360–400 nm is attributed to an intramolecular CT transition from the phenoxazine unit to the DBP segment. The experimentally observed absorption spectrum is further corroborated by time-dependent DFT (TD-DFT) calculations implementing the long-range-corrected hybrid functional ωB97XD. The range-separation parameter (ω) was nonempirically optimized in the presence of implicit solvation using the conductor-like polarizable continuum model (CPCM) with toluene as the solvent. This solvent-influenced tuning procedure yielded an optimal ω value of 0.039 Bohr^–1^. The calculations revealed that the high-energy band belongs to S_0_→S_5_ and S_0_→S_4_ transitions (Table S1). The natural transition orbitals (NTOs) of these transitions revealed the localization on the DBP unit and major localization on the phenoxazine unit with minor CT to DBT, respectively (Figure S9). The low-energy weak absorption band belongs to an S_0_→S_1_ transition, which revealed CT from the phenoxazine unit to the DBP fragment. The UV–vis absorption spectrum of the neat **DBP-PXZ** film remains largely unchanged compared to its solutions, displaying similar bands with only a modest red shift of ca. 2 nm (Figure 3a). The low-energy absorption tail slightly extended up to ca. 500 nm can be ascribed to weak electronic coupling between adjacent chromophores in the solid state.

The toluene solution of **DBP-PXZ** exhibits blue emission with a PL maxima at ca. 488 nm and a PL lifetime of 5.45 ns with the monoexponential fit of the PL decay curve (Figure 3b, Tables 2, S2). The PL spectrum of a THF solution is markedly red-shifted to a greenish-yellow range with a PL maximum at ca. 545 nm. It shows a slightly longer emission lifetime of 7.91 ns. The substantial red shift of ca. 52 nm observed upon changing the solvent from nonpolar toluene to the comparatively polar THF provides compelling evidence for the CT nature of the emissive S_1_ state. This observation can be attributed to polarity-induced stabilization of the intramolecular CT excited state, resulting in a lowered excited-state energy in polar media. Consistent with this interpretation, the photoluminescence quantum yield (PLQY) decreases from 19.3% for a toluene solution to 14.5% for a THF solution (Table 2).

**Table 2:** Photophysical Characteristics of **DBP-PXZ** ^a^

Compound	Medium	λ_*abs*_^*max*^ (nm)	λ_*PL*_^*max*^ (nm)	PLQY (%)	τ (ns)
**DBP-PXZ**	**Toluene**	318	488	19.3	5.45
	**THF**	317	545	14.5	7.91
	**Neat film**	320	488, 545	7.6	5.4, 9.1
	**1 wt % in PMMA**	-	460	13.9	4.4

a Wavelengths of UV–vis absorption maxima (λ_*abs*_^*max*^), wavelengths of PL maxima (λ_*PL*_^*max*^), PL quantum yield (PLQY), PL lifetime (τ).

#### Photophysical Properties of Dispersions of Luminophores in the Mixtures of Miscible Solvents

The steady-state PL spectra of toluene and THF solutions of **DBP-PXZ** reveal a strong sensitivity of the emission to polarity. To further examine the influence of changes in the local environment on the emission characteristics, we investigated the steady-state PL spectra under aggregation-inducing conditions. PLQYs of the solutions of **DBP-PXZ** are higher than that of the neat film, indicating the absence of aggregation-induced emission enhancement (Table 2). Nevertheless, the emission spectra of the dispersions of the compound in THF–water mixed solvents were further investigated with gradually increasing water fractions (*f*_w_ %), which progressively induced aggregation (Figure 4a,b). This strategy enables systematic modulation of both solvent polarity and the extent of aggregation, allowing direct assessment of influence of molecular packing and restricted conformational relaxation on the emissive pathways.^48^ From a pure THF solution of **DBP-PXZ** to its dispersions in intermediate water fractions (*f*_w_ of 0–60%), the emission remains dominated by a single emissive band with the gradual bathochromic shift of PL maxima from 545 to 596 nm, respectively. These modest and gradual spectral shifts can be attributed primarily to the changes in the dielectric constant of the local environment. Upon further increasing the water fraction (*f*_w_ of 70–90%), nanoaggregation became prominent, and the emission progressively blue-shifted from yellow to green and eventually cyan (PL maxima at 540–498 nm, respectively). These spectra are indicative of suppressed geometric relaxation of the CT excited state due to gradual progressive aggregation, favoring higher-energy emissive states.^49^ At the highest water fraction (*f*_w_ = 99%), **DBP-PXZ** exists predominantly in a highly aggregated state, leading to a pronounced modulation of the PL spectrum. The primary PL maxima undergo a substantial blue-shift to ca. 478 nm, indicative of higher-energy emission associated with rigidly confined molecules. Concurrently, a distinct lower-energy emissive shoulder emerges at ∼545 nm. This low-energy emission can be reasonably ascribed to excimer emission originating from densely packed molecules, where close intermolecular proximity enables the formation of relaxed intermolecular excited states. The PL evolves into a nearly white emission comprising balanced emissive high- and low-energy emissions. Such behavior is consistent with previous reports of white emission arising from the simultaneous contribution of monomeric and excimer emissions in structurally heterogeneous organic assemblies.^50^

**Figure 4:**
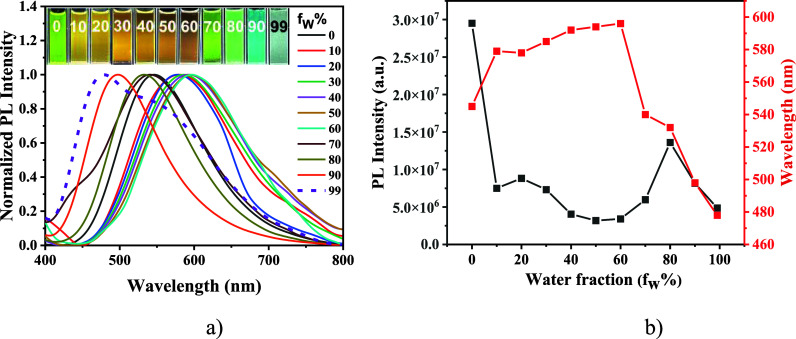
PL spectra and visual representation under UV excitation of dispersion of **DBP-PXZ** in the different THF–water mixtures (a) and impact of water fraction in the dispersion of **DBP-PXZ** in THF–water mixture on PL intensity and wavelength of PL maxima (b).

TD-DFT calculations were performed to gain molecular-level insight into the origin of the low-energy emissive component observed under highly aggregated conditions. For the isolated molecule of **DBP-PXZ**, TD-DFT calculations at the TD/ω*B97XD/6-31G** level incorporating CPCM solvation (THF) yielded an S_1_ state energy of 3.10 eV (Table S1, Figure S10). To approximate the effect of close intermolecular contacts, a representative dimeric **DBP-PXZ** assembly was optimized at the ωB97XD/6-31G** level (Figure S11), followed by excited-state calculations at the same TD-DFT level (TD/ω*B97XD/6-31G**, CPCM/THF) (Figure 5). Notably, the dimer exhibits a significantly stabilized S_1_ state at 2.93 eV, corresponding to an energetic stabilization of 0.17 eV relative to the isolated molecule (Figure 5, Table S1). This magnitude of stabilization may indicate the emergence of a low-energy emissive band arising from an intermolecular interaction induced excited state. Further insight is provided by the NTO analysis of the dimer excited state (Figure 5), which reveals pronounced intermolecular CT character. Specifically, the hole density is predominantly localized on the phenoxazine donor unit of one molecule, while the electron density is mainly distributed over the BDT acceptor unit of the neighboring molecule. This spatial separation of hole and electron densities across two molecular units provides compelling evidence for an intermolecular CT excited state, characteristic of an excimer state formed in aggregates. The neat film of **DBP-PXZ** exhibits a closely analogous dual emission, with high-energy blue and low-energy greenish-yellow emission bands concurrently giving near-white emission. The emergence of the low-energy emissive channel is consistent with intermolecularly influenced emission of the solid state. These contributions are further probed through PL analysis of the solid solutions of **DBP-PXZ** in a host This is done in the following section to elucidate the interplay between distinct emissive pathways.

**Figure 5:**
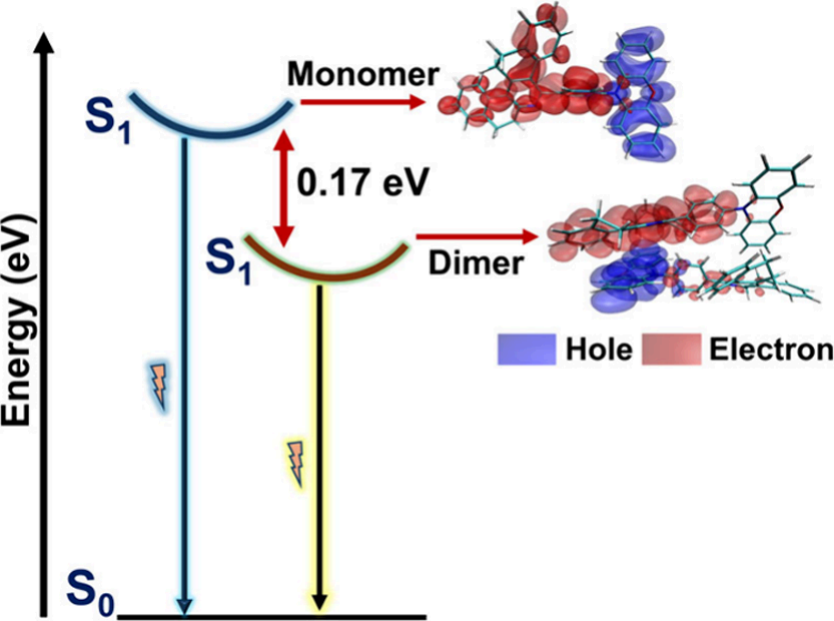
Energy level diagram based on the computed S_1_ excited state of the monomer and dimer of **DBP-PXZ** and their corresponding NTOs at ω*B97XD/6-31G**.

#### Photophysical Properties of the Neat Film and of the Solid Solution in a Polymeric Host

The neat film of **DBP-PXZ** exhibits dual emission with PL maxima at ca. 488 and 545 nm, respectively. As a result, the film emits white light, with CIE coordinates of (0.31, 0.38) (Figure 6a,c), approaching the coordinates of natural white emission (0.33, 0.33). The dual-band PL spectrum of the film can be assigned to high-energy blue emission from the intramolecular CT S_1_ state and low-energy greenish-yellow emission from the excimer CT state. A multiexponential fit to PL decay further revealed an intensity-averaged lifetime of 5.4 ns for the 488 nm band and 9.1 ns for the 545 nm band (Figure 6b, Tables 2 and S2). This observation is in line with the generally longer lifetimes expected for relaxed excimer CT states relative to those of the monomeric CT-state emission.

**Figure 6:**
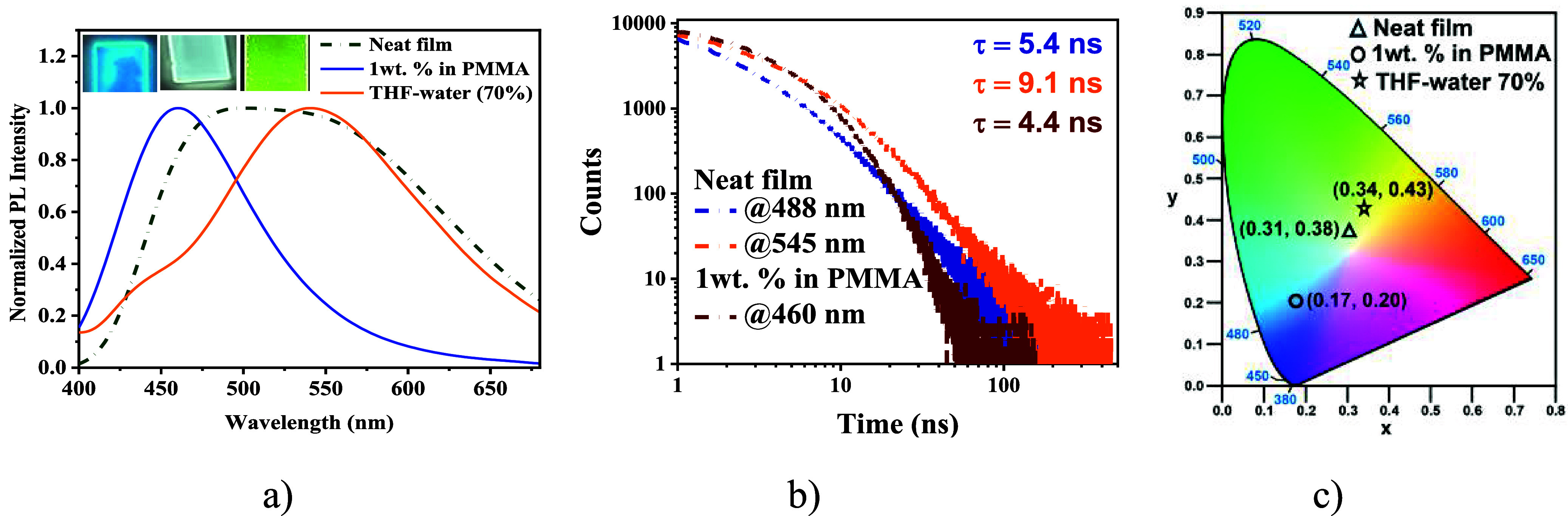
Steady-state PL spectra of neat and host-based films (a); corresponding PL decay curves (b) and CIE chromaticity coordinates (c) of neat films and of the films of a 1 wt % solid solution of **DBP-PXZ** in PMMA.

Further support for intermolecular-interaction-mediated excimer formation was gained from the investigations of photophysical properties of host–guest systems. A series of solid solutions comprising **DBP-PXZ** dispersed in the polar DPEPO host matrix were prepared with gradually increasing dopant concentrations (10–90 wt %) (Figure S12). As the concentration of **DBP-PXZ** increased, the enhanced proximity among chromophores facilitated stronger intermolecular π-interactions, thereby promoting excimer formation. Consistent with this trend, the PL spectra of the host-containing films display a progressively intensified low-energy excimer emission, characterized by a band appearing with a maximum at ca. 570 nm. Notably, this excimer emission band is red-shifted by ca. 25 nm relative to the excimer emission observed for the neat film of **DBP-PXZ** (ca. 545 nm). This bathochromic shift is readily rationalized by additional stabilization of the excimer CT state in the more polar DPEPO environment, which stabilizes the excimer excited state and causes a bathochromic shift. To isolate the intrinsic monomeric emission and fully suppress intermolecular interactions, a film of dilute (1 wt %) solid solution of **DBP-PXZ** in a PMMA was prepared. The chromophores were spatially well-separated, effectively eliminating excimer formation. The resulting film of the molecular dispersions in PMMA displayed a blue emission with a PL maximum at ca. 460 nm, accompanied by a PL lifetime of 4.4 ns and CIE coordinates of (0.17, 0.20) (Table 2, Figure 6a–c). These results are consistent with a purely monomeric intramolecular CT state. Taken together with the results observed for a neat film and for the film of the solid solution of **DBP-PXZ** in a DPEPO host, these findings unambiguously demonstrate that **DBP-PXZ** supports two distinct emissive excited states, i.e., a high-energy monomeric CT state emission and a lower-energy excimer CT state emission. This dual-state emission underscores the versatility of the compound and highlights its potential for single-component host-containing blue and white host-free OLED applications.

In air, a film of neat **DBP-PXZ** showed a PLQY of 7.6%. This value is lower than those of both toluene and THF solutions, which can be explained by excitonic distribution into two distinct emissive states that are monomeric and an excimer state, causing white emission (Table 2). Each state has radiative and nonradiative relaxations with a PLQY of merely 7.6%. The PLQY value of 13.9% observed for the film of PMMA molecularly doped with **DBP-PXZ** was also evidence of high excitonic recombination from one emissive pathway, i.e., from the monomeric CT state. The comparably low PLQY of the neat film is attributed to the distribution of excitons into two distinct pathways.

### Charge-Injecting and Charge-Transporting Properties

To emphasize the charge-injecting capabilities of the film of **DBP-PXZ**, photoelectron emission (PE) spectroscopy was used to determine the ionization energy (IE_PE_) and electron affinity (EA_PE_). Energy levels of solid films are crucial for the design of electroluminescent devices. An IE_PE_ of 6.0 eV was observed (Figure S6), accompanied by an EA_PE_ of 3.05 eV determined using EA_PE_ = IE_PE_ – *E*_g_^opt^, where *E*_g_^opt^ is the optical bandgap determined from the onset energy of UV–vis absorption spectra of the neat film of **DBP-PXZ (** Figure 3a). Analyzing the obtained values of IE_PE_ of 6.0 eV and EA_PE_ of 3.05 eV, it is evident that either hole-injecting or hole-transporting layers should be used between the anode (ITO with a work function of 4.7 eV) and the layer of **DBP-PXZ** for the reduction of the energy barrier between the ITO and **DBP-PXZ**. Electron injection from the cathode (e.g., LiF/Al with the work function of 2.9 eV) into the **DBP-PXZ** layer is predicted to be efficient due to the negligible energy barrier between **DBP-PXZ** and LiF/Al.

The charge-transporting properties of **DBP-PXZ** were examined according to the time-of-flight (TOF) methodology.^51,52^ The layer of **DBP-PXZ**, with the thickness (*d*) of 2.592 μm, was deposited in a vacuum on an ITO-covered glass substrate (Figure S7). By top Al electrode deposition, the TOF sample with the structure ITO/**DBP-PXZ**/Al was completed. Upon excitation of the layer of **DBP-PXZ** by a laser pulse with the emission wavelength of 355 nm through ITO, charges were generated near the ITO/**DBP-PXZ** interface. Selectively, holes or electrons moved through the layer of **DBP-PXZ** when positive or negative voltages (V) were applied to ITO, respectively. Transit times (*t*_tr_) of holes or electrons were used to calculate hole (μ_h_) and electron (μ_e_) mobility values at the different electric fields using the formula μ = *d* ^2^/*Vt*_tr_. The *t*_tr_ values for holes and electrons were determined from the slope intersections of the current transit curves plotted on log–log scales (Figure S8).

**DBP-PXZ** showed very balanced hole and electron transport over a wide range of electric fields. For example, at the electric field (*E*) of 7.4·10^5^ V/cm, μ_h_ of 1.17·10^–5^ cm^2^/(V s) and μ_e_ of 2.33·10^–5^ cm^2^/(V s) were recorded (Figure 7). Such charge balance is beneficial for OLED emitters.^53^ It should be noted that electron mobility is slightly higher than hole mobility at the same electric fields. At an *E* higher than 1 × 10^6^ V/cm, μ_e_ values reached 1.8 × 10^–4^ cm^2^/(V s). The analysis of hole and electron mobilities as the Poole–Frenkel function of electric fields revealed a relatively high field effect parameter βs of 7.2 × 10^–3^ cm/V and 8.9 × 10^–3^ cm/V for holes and electrons, respectively. As a result, the low zero field hole and electron mobility values of 2.3 × 10^–8^ V/cm and μ_h_ of 1.2 × 10^–8^ cm^2^/(V s), respectively, were obtained. Such dependences of charge mobility values are expected for charge-transporting materials with high dipole moments.^54,55^ Charge-transporting materials with high dipole moments, such as **DBP-PXZ**, have limited potential for use as OLED hosts.^56^ **DBP-PXZ** as an OLED emitter may require selecting an appropriate host.

**Figure 7:**
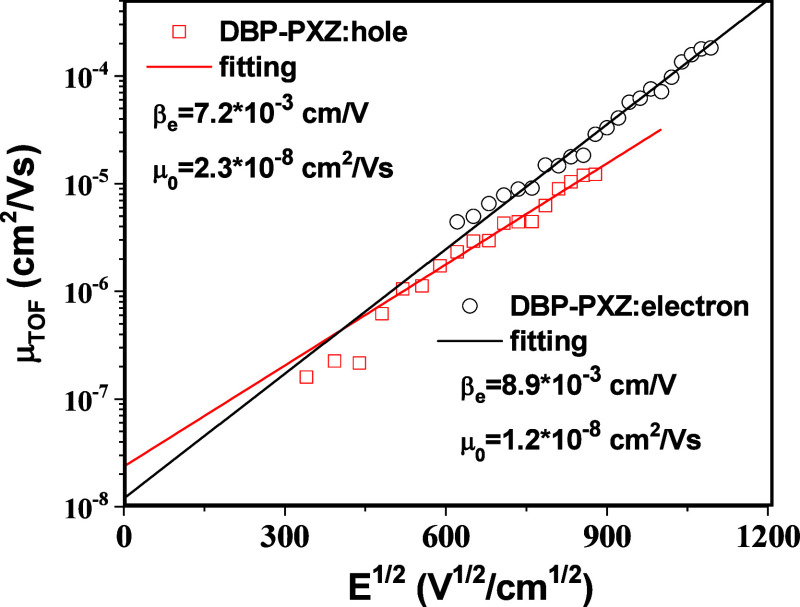
Plots of hole and electron mobilities of **DBP-PXZ** versus electric field.

### Electroluminescent Devices

Taking into account the white emission and bipolar charge transport of **DBP-PXZ**, we aimed to investigate whether the single-component dual emission of **DBP-PXZ** originating from monomeric CT and excimers enables the direct fabrication of a host-free white electroluminescent device depositing merely the layer of **DBP-PXZ** as the emissive layer. In addition, dilution of **DBP-PXZ** in a wide-bandgap host effectively suppresses intermolecular interactions, confining exciton recombination to the monomeric CT state and yielding pure blue electroluminescence (EL), thereby enabling emission tunability from a single molecular system. To clarify these expectations, host-free and host-containing OLEDs with the emitter **DBP-PXZ** were studied using the device structure of ITO/HAT-CN (5 nm)/NPB (40 nm)/mCBP (8 nm)/EML (30 nm)/TSPO1­(4 nm)/TPBi (40 nm)/LiF/Al (Figures 8a, S13). In this structure, the layers of 1,4,5,8,9,11-hexaazatriphenylene­hexacarbonitrile (HAT-CN) and *N*,*N*′-di­(1-naphthyl)-*N*,*N*′-diphenyl-(1,1′-biphenyl)-4,4′-diamine (NPB) serve as the hole-injection and hole-transport layers, respectively. A thin layer of 3,3′-di­(9*H*-carbazol-9-yl)-1,1′-biphenyl (mCBP) was introduced as an exciton-blocking layer to suppress exciton diffusion toward the hole-transporting region. Diphenyl­[4-(triphenylsilyl)­phenyl]­phosphine oxide (TSPO1) and 2,2′,2″-(1,3,5-benzenetriyl)-tris­(1-phenyl-1*H*-benzimidazole) (TPBi) functioned as the hole-blocking and electron-transport materials, respectively. Three devices (ES1–ES3) with distinct emissive layers were fabricated.

**Figure 8:**
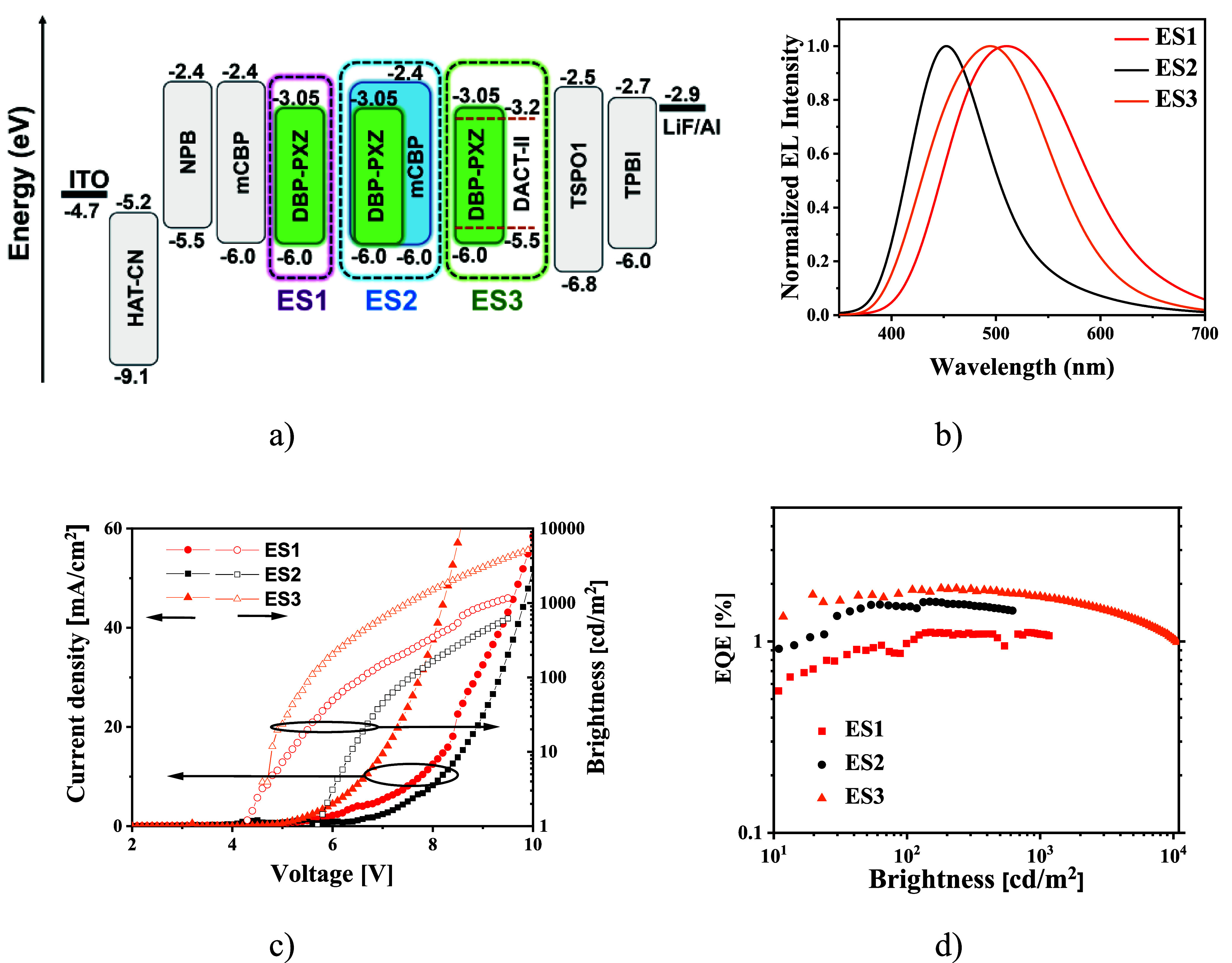
Energy level diagram of devices with three distinct emissive layers (a), normalized EL spectra at 10 V (b), the plots of current density and brightness vs voltage (c), and brightness–external quantum efficiency characteristics of ES1–ES3 (d).

In the fabrication of device ES1, the layer of **DBP-PXZ** was deposited as the host-free EML. Device ES1 exhibited a broad EL spectrum spanning the range of ca. 385–800 nm, with CIE coordinates of (0.278, 0.409) (Figure 8b, Table 3). Nevertheless, the EL spectrum remained essentially invariant with increasing applied voltage (Figure S14a). This observation indicates that the relative contributions of the monomeric CT and excimer emissive states are dictated by intrinsic excited-state energetics rather than by field-induced exciton redistribution. This statement is also supported by the relatively high full width at half-maximum (fwhm) of 145 nm estimated for device ES1. To weigh the contributions of the monomeric CT and excimer emissive states, we fitted normalized EL spectra of devices ES1–ES3 with two peaks using the Gaussian model (Figure S15). The cumulative spectra closely matched the recorded EL spectra of devices ES1–ES3, with low fitting errors (*R* ^2^ > 0.997). According to the fitting results, the contribution of excimer emissive states was found to be higher than that of monomeric CT states. The area under the peak fit 2 (ca. 91.8) was larger than that of peak fit 1 (ca. 60.7). Device ES1 displayed a turn-on voltage of 3.9 V. It delivered a peak external quantum efficiency (EQE) of 1.11% (Figure 8c,d). The relatively high EQE (taking into account the relatively low PLQY of 7.6% of the neat film of **DBP-PXZ**) of device ES1 is mainly attributed to the balanced hole and electron transport of the host-free EML **DBP-PXZ**. In device ES2, the **DBP-PXZ** was doped at 10% into the mCBP host. It exhibited a comparatively narrow-band blue EL spectrum with EL maxima at ca. 450 nm and CIE coordinates of (0.173, 0.166) (Figures 8b, S14D). An fwhm of 89 nm was obtained for the EL spectrum of device ES2. This spectral profile confirms the selective harvesting of the blue-emissive intramolecular CT excited state of **DBP-PXZ**, enabled by effective suppression of intermolecular interactions in the dilute solid solution in the host matrix. Nevertheless, the EL spectrum of ES2 exhibited long tails with low intensities. These tails are attributed to the excimer emissive states. For instance, the area under peak fit 2 (ca. 31.7) was smaller than that of peak fit 1 (ca. 70.8), indicating that excimer state emission should not be eliminated from the electroluminescence of **DBP-PXZ** molecularly dispersed in the low-polarity host mCBP. Compared with ES1, device ES2 showed a higher turn-on voltage of 5.3 V. This observation can be explained by the higher electron affinity of mCBP (2.4 eV) than that of **DBP-PXZ** (3.05 eV) (Figure 8a). In addition, the hole–electron balance of **DBP-PXZ** is better than that of the 10% solid solution of **DBP-PXZ** in mCBP. Compared with ES1, the slightly enhanced maximum EQE of ES2 (1.61%) can be attributed to the improved exciton confinement within the mCBP host and the intrinsically higher PLQY of the monomeric blue CT emission. Device ES3 contains **DBP-PXZ** as the primary emissive component, with a low co-deposition of DACT-II (3 wt %) to modulate charge balance and exciton recombination within the emissive layer. The resulting EL spectrum exhibits an EL maximum at ca. 500 nm with CIE coordinates of (0.235, 0.382) without perturbing much the intrinsic emission balance of **DBP-PXZ**. According to the fitting results shown in Figure S15, the contribution of excimer emissive states is higher than that of monomeric CT states because the area under peak fit 2 (ca. 91.6) is larger than that of peak fit 1 (ca. 47.9). The trend of fitted peaks of the EL spectrum of device ES3 is close to that of device ES1. A slightly narrower EL spectrum recorded at 10 V with an fwhm of 134 nm was obtained for device ES2 in comparison to that of device ES1. Device ES3 showed the best overall performance, achieving a maximum EQE of 1.9% and a peak luminance exceeding 10000 cd/m^2^ (Table 3). This result indicates that a low concentration of DACT-II allows enhancing device efficiency without significantly perturbing the intrinsic emission balance of **DBP-PXZ**.

**Table 3:** EL Parameters of Devices ES1–ES3

Device	Emissive layer	*V*_on_ (V)	Maximum brightness (cd/m^2^)	Current efficiency (cd/A)	Power efficiency (lm/W)	EQE (%)	Wavelength of EL maxima (nm)	fwhm (nm)	CIE coordinates (*x*, *y*)
**ES1**	**DBP-PXZ**	3.9	1219	2.81	1.27	1.11	510	145	0.278, 0.409
**ES2**	**DBP-PXZ** (10%):mCBP	5.3	619	1.99	0.85	1.61	450	89	0.173, 0.166
**ES3**	DACT-II (3%):**DBP-PXZ**	4.6	10632	4.59	2.73	1.90	500	134	0.235, 0.382

## 4 Conclusions

We synthesized and investigated a D−π–A type luminophore, having a phenoxazine donor moiety and a tetrahydrodibenzophenanthridine acceptor fragment linked through a twisted phenylene bridge. The compound manifests controllable dual-emission within a single-component molecular system. The electron-rich phenoxazine donor coupled with the acene-like tetrahydrodibenzo­phenanthridine acceptor via a partially twisted phenylene bridge enables a delicate balance between intramolecular charge-transfer and intermolecular interactions, giving rise to dual emission bands. Consequently, the neat film of the newly synthesized compound exhibits white emission with CIE coordinates of (0.31, 0.38). The spectral analysis of the film of the molecular mixture of the compound with a host corroborates the high-energy blue emission from the monomeric charge-transfer state and the low-energy greenish-yellow band from the dimeric charge-transfer state. Exploiting these distinct dual-emissive channels, host-free OLEDs exhibit broad electroluminescence spanning the range of ca. 385–800 nm. The controlled dilution in a wide-bandgap host selectively yields pure blue emissive devices with the intensity maxima of electroluminescence at ca. 450 nm. This work establishes a robust strategy for engineering of a single molecular framework with tunable multiemissive channels and underscores the potential of molecular-level control to comprehend structurally simple yet spectrally versatile OLEDs.

## Supplementary Material



## Notes

Detailed description and characterization of the synthesis, additional electrooptical, computational, and photophysical characteristics, and detailed electroluminescent device characteristics (PDF)
