# Supplementary material for: Molecularly Engineered Dual-Emission Pathways with Monomer–Excimer Interplay for Single-Component Blue and White Organic Light-Emitting Diodes
Source: ACS Appl Opt Mater. 2026 May 27;4(6):1787–97. doi: 10.1021/acsaom.6c00161 (PMC13317630; doi:10.1021/acsaom.6c00161)
Supplement: Supplementary file 1 [file ot6c00161_si_001.pdf]

Supporting Information

Molecularly Engineered Dual-Emission Pathways with Monomer-Excimer Interplay for Single-Component Blue and White Organic Light-Emitting Diodes

Ehsan Ullah Rashid[1], Rishika Suresh[2], Dmytro Volyniuk[1], Sathiyanarayanan Kulathu Iyer[2*], Juozas V. Grazulevicius[1*]

[1]*Department of Polymer Chemistry and Technology, Faculty of Chemical Technology, Kaunas University of Technology, K. Baršausko g. 59, LT-51423, Kaunas, Lithuania*

[2]*School of Advanced Sciences, Vellore Institute of Technology University, Vellore, India*

* Corresponding author.
E-mail address: sathiyanarayanank@vit.ac.in (S. Kulathu Iyer), juozas.grazulevicius@ktu.lt (Juozas V. Grazulevicius).

Content

1. Synthesis and characterization………………………………………….…S2

2. Electrooptical characteristic…………..……………………………………. S6

3. Computational characteristics…………..………………………………… S7

4. Photophysical characteristics…………..……………………………...... S8

5. Electroluminescent devices section……………….……………………... S10

1. Synthesis and characterization

Synthesis of 4-(10H-phenoxazin-10-yl)benzaldehyde:

To a stirred solution of phenoxazine (1 eq. 1 g) in 10 ml of dimethyl formamide (DMF) at room temperature, potassium carbonate (2 eq. 1.49 g), CuI (0.2 eq. 209 mg), 4-bromo-benzaldehyde (1 eq. 1 g) was added. Then the reaction mixture was heated at 100^0C for 24 h under nitrogen atmosphere. After completion of the reaction (monitored by TLC), the mixture was cooled to room temperature, ice was added followed by an aqueous solution of sodium thiosulphate. The mixture was stirred for 15 min. The solution was filtered and concentrated under reduced pressure. The crude product was purified by column chromatography (n-hexane : ethyl acetate ; 9:1) using silica gel (100-200 mesh) to obtain a pale white colour solid with 43 % yield.

^{1}H NMR (400 MHz, $CDCl_3$) δ: 10.10 (s, 1H), 8.12-8.10 (d, 2H, J = 8 Hz), 7.56-7.54 (d, 2H, J = 8 Hz), 6.74-6.67 (m, 4H), 6.63-6.59 (m, 2H), 5.97-5.95 (q, 2H).

^{13}C NMR (100 MHz, $CDCl_3$) δ: 191.03, 145.02, 144.09, 135.88, 133.50, 132.31, 131.46, 123.32, 122.06, 115.83, 113.43.

Synthesis of 10-(4-(7,8,13,14-tetrahydrodibenzo[a,i]phenanthridin-5-yl)phenyl) -10H-phenoxazine:

To a stirred solution of 4-(10*H*-phenoxazin-10-yl)benzaldehyde (1 eq. 600 mg) in 12 ml of ethanol, ammonium acetate (1.5 eq. 231 mg) was added. The reaction mixture was slightly warmed and 3,4-dihydronaphthalen-2(1*H*)-one (2 eq. 584 mg) was added. The reaction mixture was heated for about 15 minutes and kept stirring at room temperature for 24 h. Once the reaction was completed (confirmed by TLC), the reaction mixture was separated using ethyl acetate and the organic layer was evaporated under reduced pressure to give dark yellow crude. The crude product was purified by column chromatography (n-hexane : ethyl acetate; 16:1) using silica gel (100-200 mesh) to obtain a pale-yellow colour solid with 10 % yield. The melting point was 235 – 238 ^{o}C.

^{1}H NMR (400 MHz, $CDCl_3$) δ: 7.68-7.66 (d, 2H, J = 8 Hz), 7.55-7.53 (m, 2H), 7.39-7.27 (m, 6H), 7.18-7.14 (m, 1H), 6.95-6.89 (m, 2H), 6.70-6.61 (m, 6H), 6.07-6.05 (m, 2H), 3.20-3.12 (m, 4H), 3.01-2.98 (m, 2H), 2.82-2.79 (t, 2H, J = 6 Hz).

^{13}C NMR (100 MHz, $CDCl_3$) δ: 158.30, 152.92, 143.98, 139.69, 138.97, 138.32, 134.29, 132.89-132.71, 130.80, 129.55 129.21, 129.05, 128.75, 127.95, 127.87, 127.32, 127.13, 126.14, 125.42, 123.22, 121.31, 115.43, 113.40, 33.25, 29.54-29.45, 29.18.

Mass spectrum (m/z): calculated 540.67, found 540.

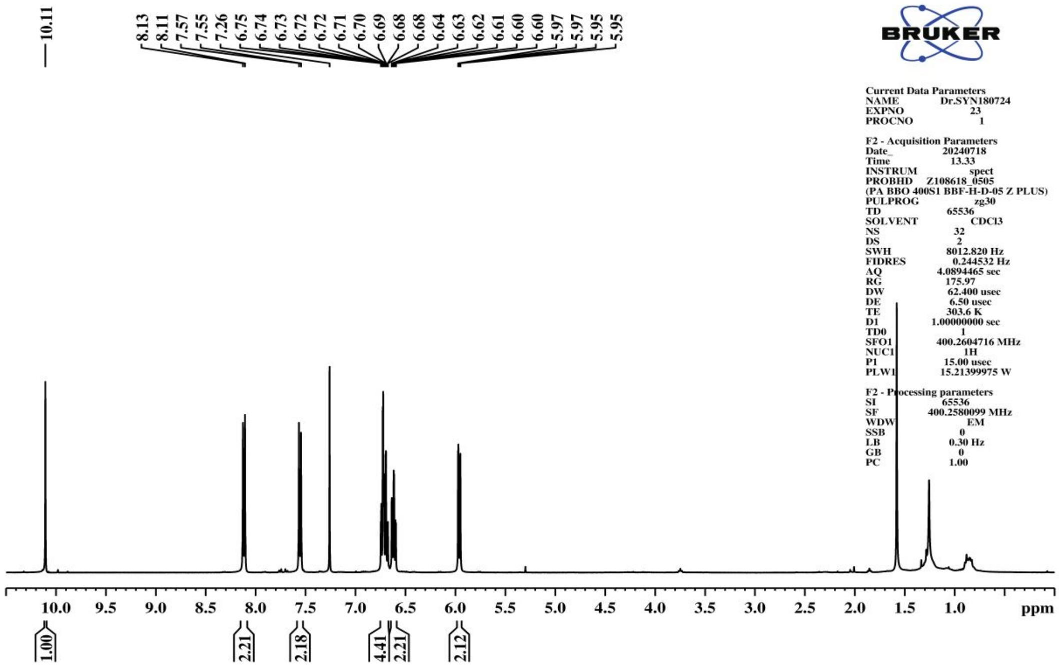

Figure S1. ^{1}H NMR spectrum of 4-(10H-phenoxazin-10-yl)benzaldehyde.

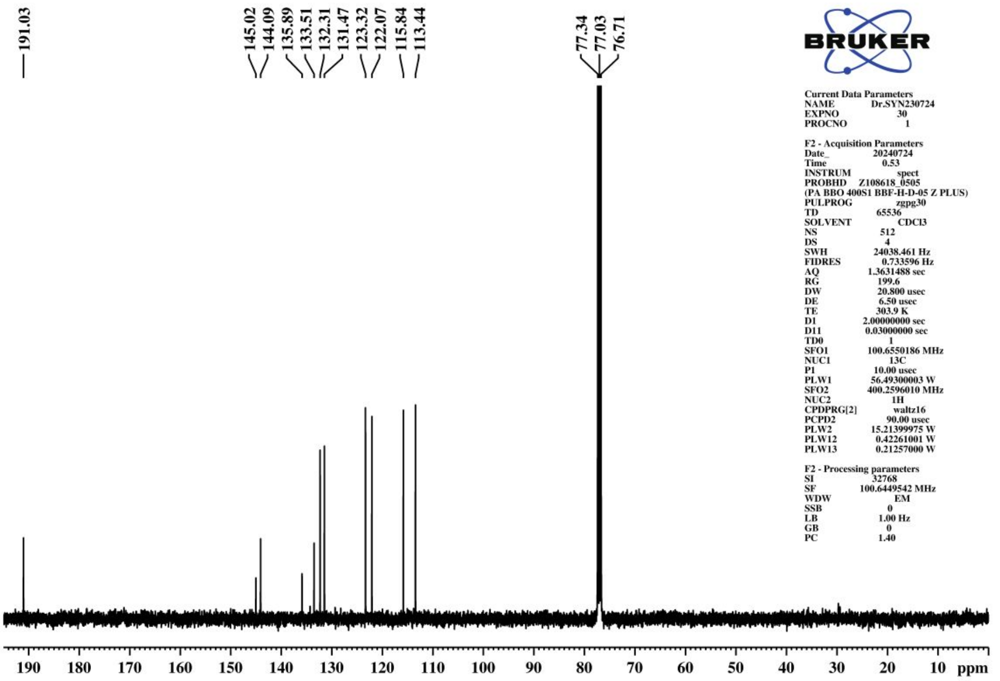

Figure S2. ^{13}C NMR spectrum of 4-(10H-phenoxazin-10-yl)benzaldehyde.

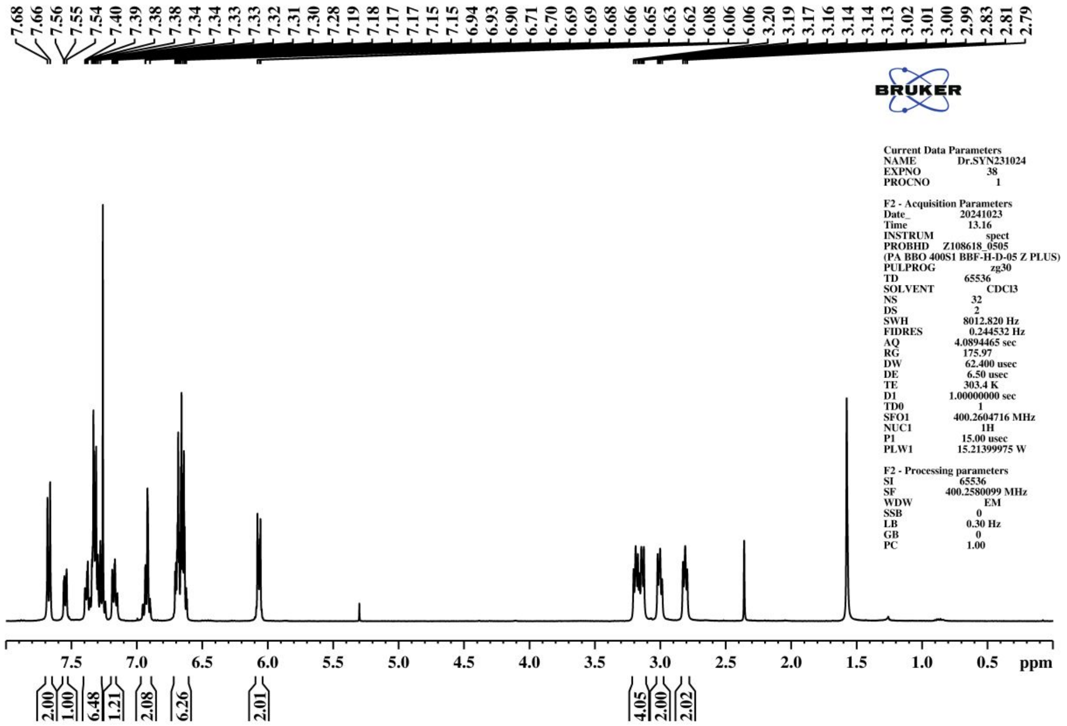

Figure S3. ^{1}H NMR spectrum of 10-(4-(7,8,13,14-tetrahydrodibenzo[a,i]phenanthridin-5-yl)phenyl)-10H-phenoxazine.

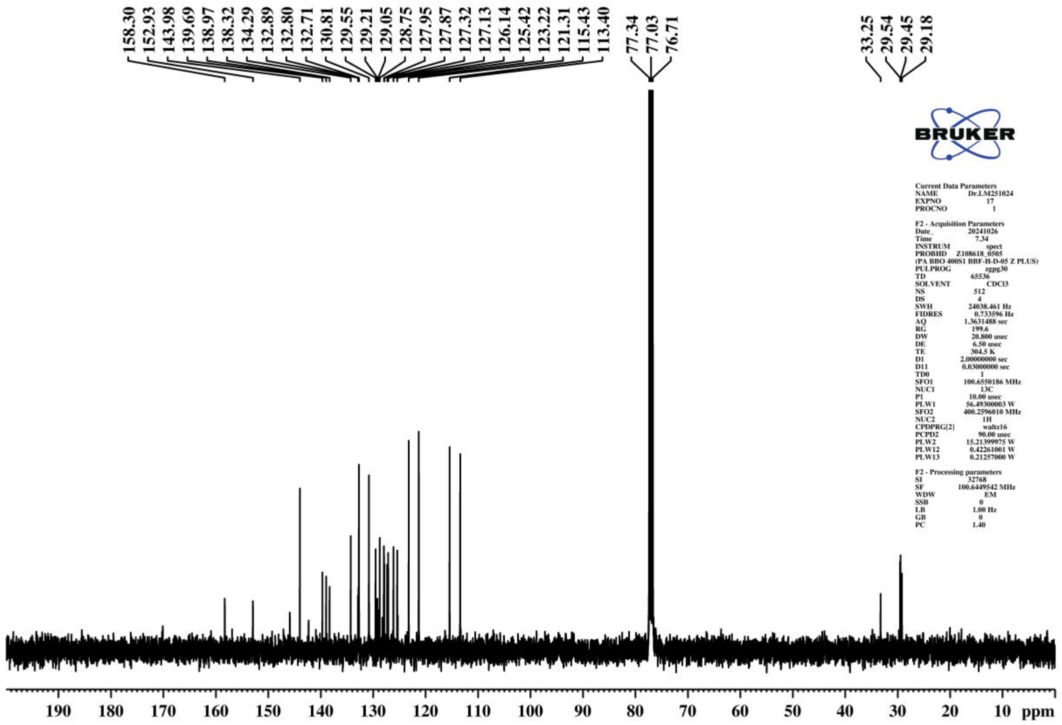

Figure S4. ^{13}C NMR spectrum of 10-(4-(7,8,13,14-tetrahydrodibenzo[a,i]phenanthridin-5-yl)phenyl)-10H-phenoxazine.

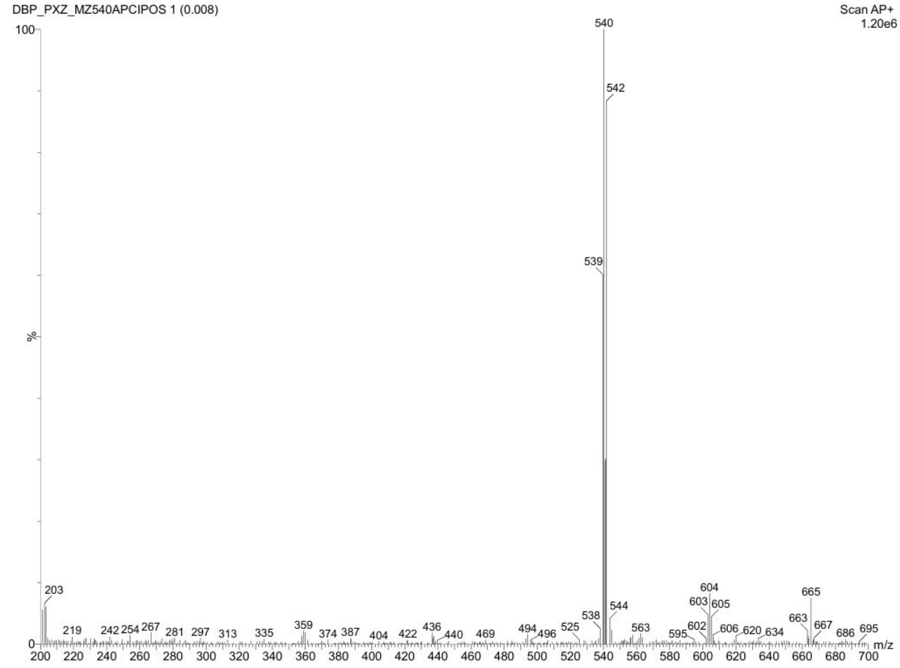

Figure S5. Mass spectrum of 10-(4-(7,8,13,14-tetrahydrodibenzo[a,i]phenanthridin-5-yl)phenyl)-10H-phenoxazine.

2. Electrooptical characteristic

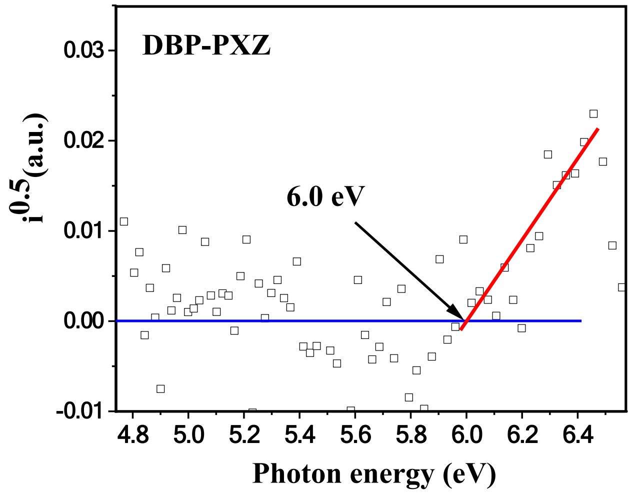

Figure S6. PE spectrum of the film of **DBP-PXZ.**

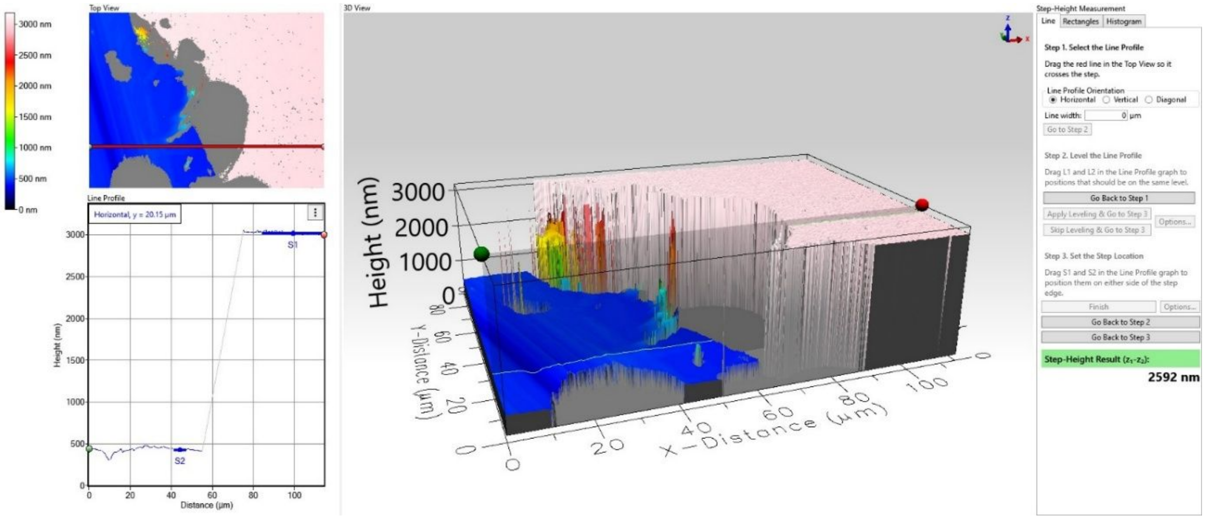

Figure S7. The thickness measurements of the TOF sample of **DBP-PXZ**.

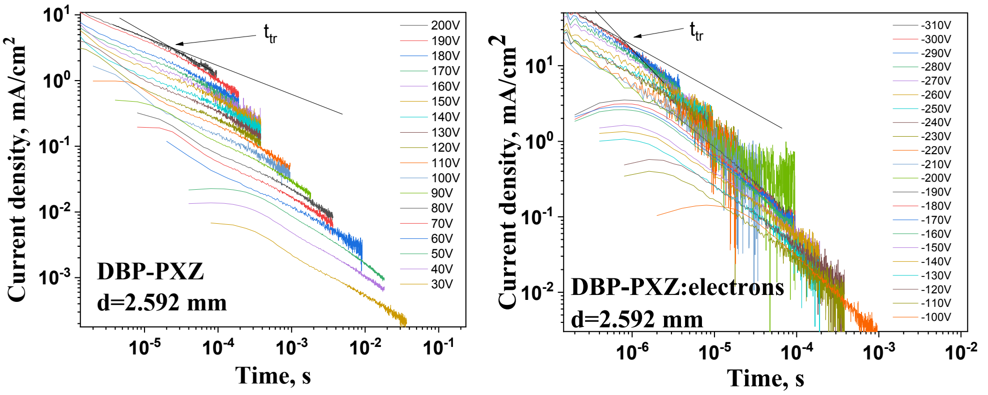

Figure S8. TOF signals for holes and electrons.

3. Computational characteristics

Table S1. TD-DFT characteristics of **DBP-PXZ** monomer and dimer at TD/ω*B97XD/6-31G**.

Molecule	Solvation	State	Energy (eV)	Wavelength (nm)	Oscillator strength (*f*)	Excitation Nature
**DBP-PXZ (Monomer)**	CPCM/toluene	S_1	3.09	400	0.003	CT
		S_4	3.94	314	0.080	LE_{PXZ} + CT
		S_5	3.98	311	0.476	LE_{DBP}
	CPCM/THF	S_1	3.10	399	0.003	CT
**DBP-PXZ (Dimer)**	CPCM/THF	S_1	2.93	422	0.007	CT

Abbreviations: phenoxazine (PXZ), tetrahydrodibenzophenanthridine (DBP).

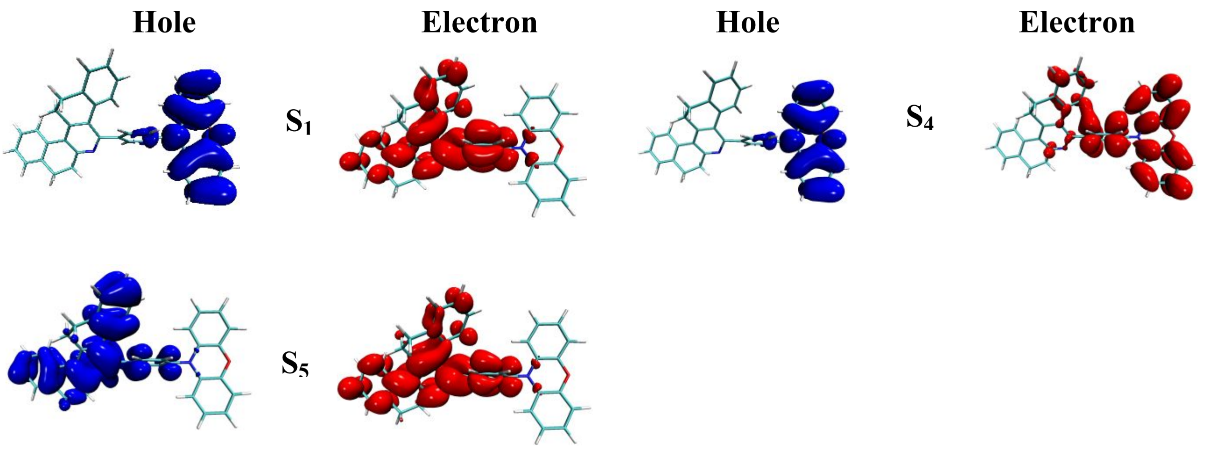

Figure S9. NTOs of S_1, S_4 and S_5 excited states of **DBP-PXZ** at TD/ω*B97XD/6-31G** and CPCM/toluene solvation.

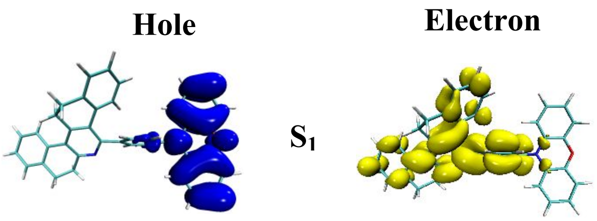

Figure S10. NTOs of S_1 excited state of **DBP-PXZ** at TD/ω*B97XD/6-31G** and CPCM/THF solvation.

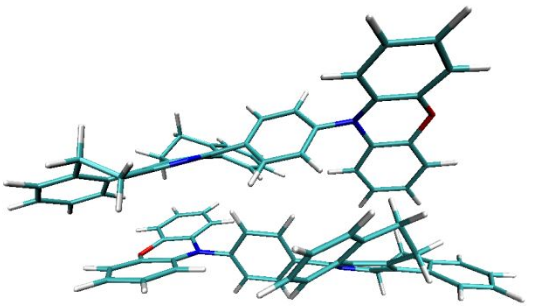

Figure S11. Optimized geometry of the dimer of **DBP-PXZ** at ωB97XD/6-31G** level.

4. Photophysical characteristics

The PL decay curves have been fitted using single and multi-exponential components as per eq. (**S1**)

$$I(t) = \sum_{i=1}^{n} A_i e^{\left(\frac{-t}{\tau_i}\right)} \qquad \textbf{(S1)}$$

Where $I(t)$ denote the fluorescence intensity as a function of time, normalized relative to its value at 0. The n is number of exponential components. The τ_i and A_i are the emission lifetime and amplitude, respectively, of ith component. The intensity-averaged PL decay lifetime was determined according to eq. (**S2**) [9].

$$\langle\tau\rangle_{avg} = \frac{\sum_{i=1}^{n} A_i\tau_i^2}{\sum_{i=1}^{n} A_i\tau_i} \qquad \textbf{(S2)}$$

The intensity-averaged lifetime accounts for the relative contribution of each decay component (τ_i) by weighting it according to its corresponding intensity. The $\langle\tau\rangle_{avg}$ in above equation is the average PL lifetime. The quality of the fit was assessed using chi-square (χ^2) parameter, as defined in eq. (**S3**).

$$x^2 = \sum_{k=1}^{n} \frac{[N(t_k) - N_C(t_k)]^2}{N(t_k)} \qquad \textbf{(S3)}$$

This statistical measure quantifies the deviation between the experimental data and the fitted curve, thereby providing an estimate of the goodness-of-fit. Here, the discrepancy between the experimental decay function ($N(t_k)$), and the calculated decay function ($N_C(t_k)$) are evaluated over n data points. A reduced χ^2 close to 1 reflects an accurate repetition of the system by the fit, whereas values exceeding 1.2 suggest that the model does not adequately capture the experimental behaviour.

Table S2. Fitting data of PL decay toluene and THF solutions, neat film of **DBP-PXZ** and 1wt. % of **DBP-PXZ** in PMMA.

Parameters	Toluene Solution	THF Solution	Neat Film (@488nm)	Neat Film (@545nm)	1 wt.% in PMMA
**A_1**	10457.11	10259.8	7971.50	6954.18	8736.80
τ_1,	4.45	7.91	1.34	2.20	3.12
**A_2**	-	-	2720.25	2926.45	1540.86
τ_2,	-	-	4.75	8.09	7.49
**A_3**	-	-	192.77	142.66	-
τ_3,	-	-	19.30	35.17	-
$\sum A_i\tau_i^2$	-	-	1.4E+05	4.01E+05	1.7E+05
$\sum A_i\tau_i$	-	-	27323.4	43991.5	38799.8
**Δτ (ns)**	4.45	7.91	5.4	9.1	4.4
χ^2	1.005	1.003	1.106	1.001	1.002

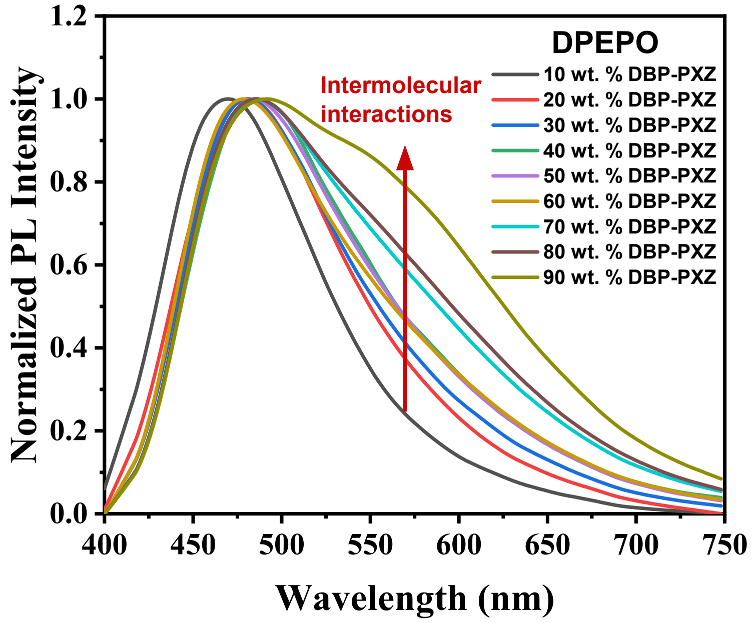

Figure S12. PL spectra of the films of 10-90 wt. % molecular dispersions of **DBP-PXZ** in DPEPO.

5. Electroluminescent devices section

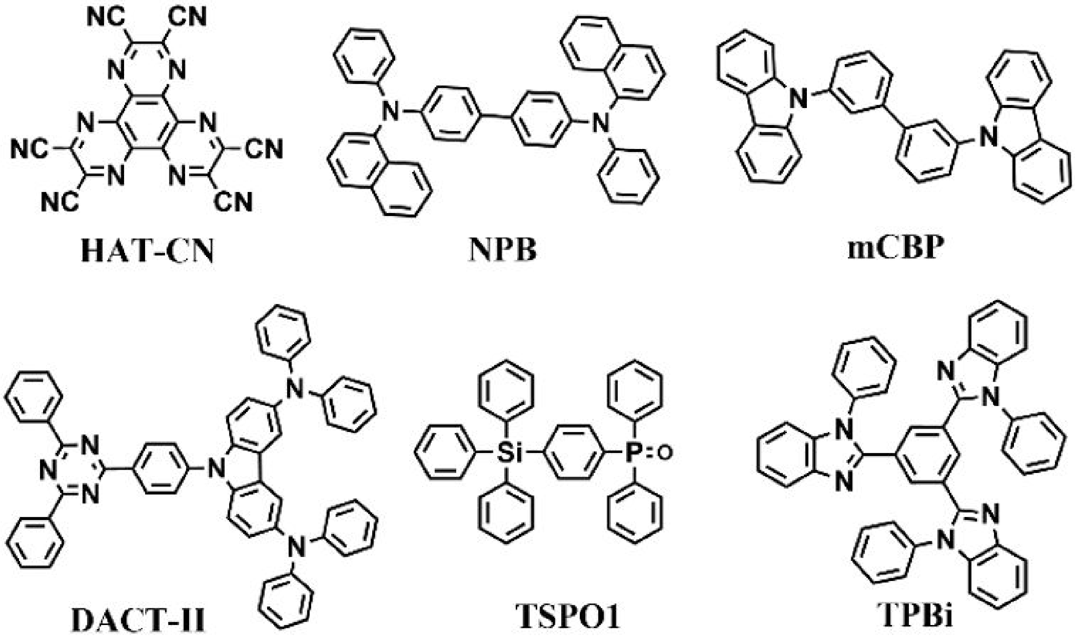

Figure S13. Chemical structure of functional layers of the devices.

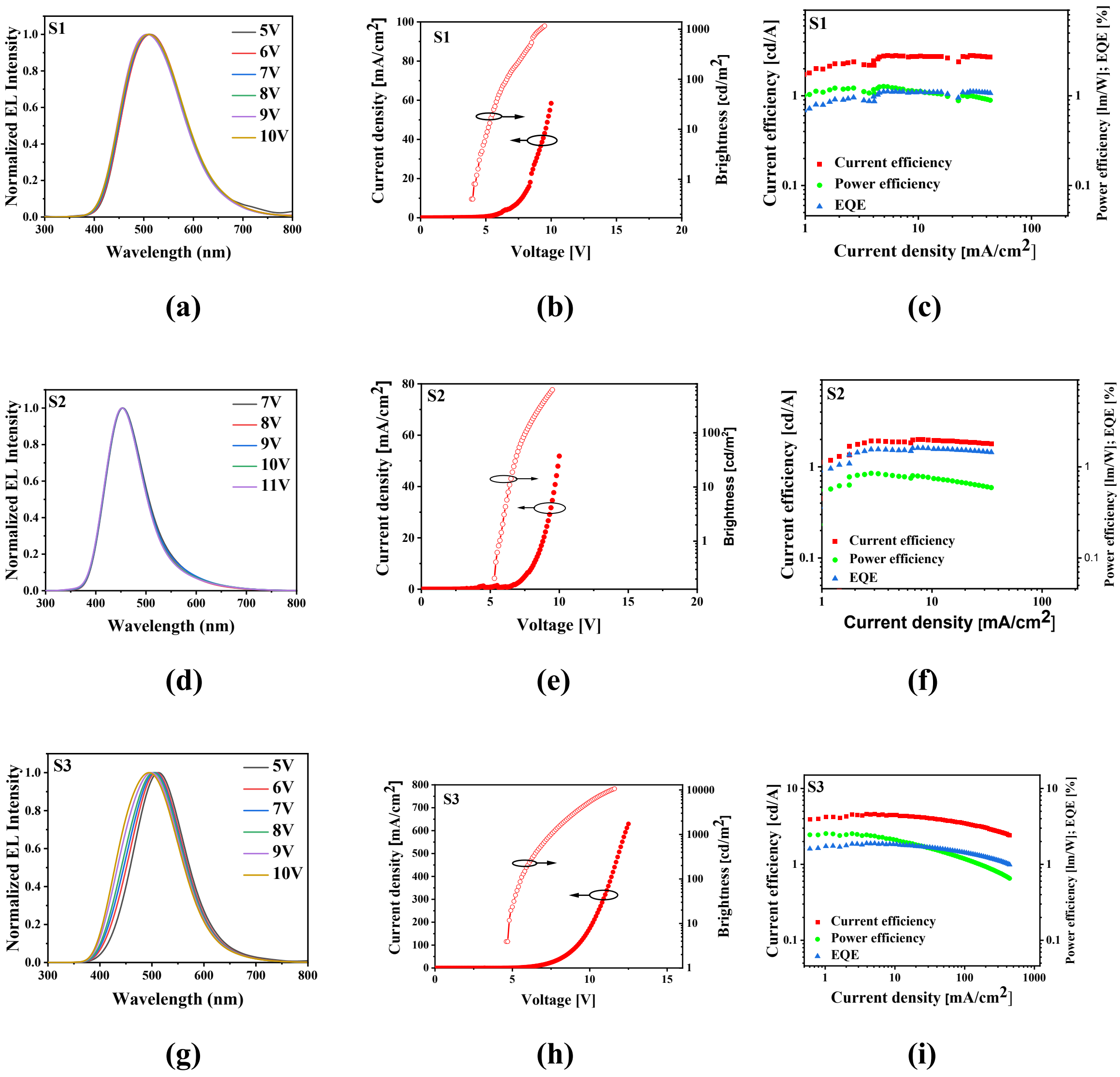

Figure S14. Normalized EL spectra, the plots of current density and brightness vs voltage, and current efficiency–current density–power efficiency–external quantum efficiency characteristics of ES1 (a-c), ES2 (d-f) and ES3 (g-i), respectively.

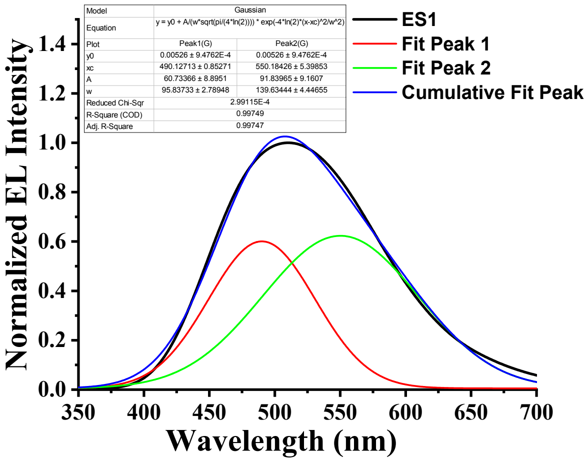

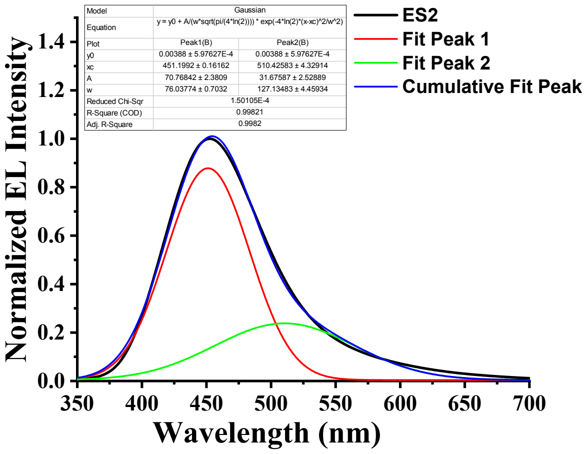

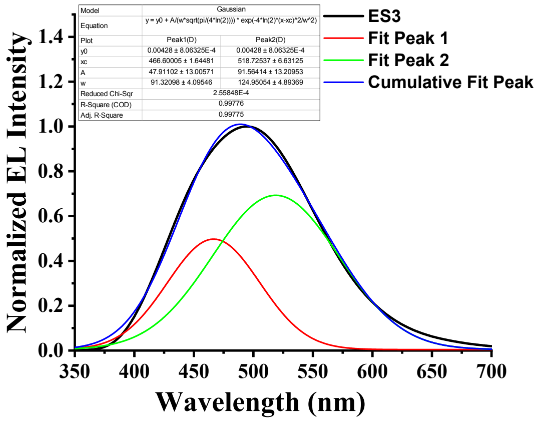

Figure S15. Multipeak fitting of normalised EL spectra of devices ES1-E3.
